# GSS-RiskAsser: A Multi-Modal Deep-Learning Framework for Urban Gas Supply System Risk Assessment on Business Users

**DOI:** 10.3390/s21217010

**Published:** 2021-10-22

**Authors:** Xuefei Li, Liangtu Song, Liu Liu, Linli Zhou

**Affiliations:** 1 Hefei Institute of Intelligent Machines, Hefei Institutes of Physical Science, Chinese Academy of Sciences, Hefei 230031, China; ltsong@iim.ac.cn (L.S.); linlizhou@iim.ac.cn (L.Z.); 2 University of Science and Technology of China, Hefei 230027, China; liuliu66@mail.ustc.edu.cn; 3 College of Environment and Energy Engineering, Anhui Jianzhu University, Hefei 230601, China

**Keywords:** natural gas supply system risk assessment, multi-modal fusion, deep learning, cross-attention mechanism

## Abstract

Gas supply system risk assessment is a serious and important problem in cities. Existing methods tend to manually build mathematical models to predict risk value from single-modal information, i.e., pipeline parameters. In this paper, we attempt to consider this problem from a deep-learning perspective and define a novel task, Urban Gas Supply System Risk Assessment (GSS-RA). To drive deep-learning techniques into this task, we collect and build a domain-specific dataset GSS-20K containing multi-modal data. Accompanying the dataset, we design a new deep-learning framework named GSS-RiskAsser to learn risk prediction. In our method, we design a parallel-transformers Vision Embedding Transformer (VET) and Score Matrix Transformer (SMT) to process multi-modal information, and then propose a Multi-Modal Fusion (MMF) module to fuse the features with a cross-attention mechanism. Experiments show that GSS-RiskAsser could work well on GSS-RA task and facilitate practical applications. Our data and code will be made publicly available.

## 1. Introduction

The advantages and the increasing demand of natural gas for urban life and industry has led to the rapid growth of the buried gas pipeline network [1]. However, rupture of the gas supply system and subsequent accidents pose a huge threat to urban public safety [2]. Due to the characteristics of natural gas, as well as the complexity of the gas supply system network in the urban environment, slight damage to the pipeline network may cause serious hazard chain effects, resulting in immeasurable disasters, deaths, casualties and property losses [3,4]. In this case, some works start to focus on dealing with the gas supply system risk problem by vulnerability assessment in the urban gas supply system network, which aims to predict the susceptibility of a community to a hazard and the prevailing condition, including physical, social–economic and political factors [5]. In this way, these systems could effectively discover systemic weakness, to provide scientific guidance for management decision-making and reduce the possibility of pipeline rupture accidents [6].

To efficiently assess the gas supply system risk, existing methods attempt to build mathematical models to predict risk possibility in cities [7,8]. In these methods, researchers prefer to consider lots of factors related to gas supply system that might cause pipeline rupture, e.g., pipeline length [9], materials [10], geometry [11], consequence level [12] and so on. Among these gas supply system risk assessment systems, most of them pay more attention to how to make the assessment model take these factors into account simultaneously. To achieve this, they manually provide various weight combinations for these different types of information [10].

However, this strategy largely relies on expert consensus that might lead to various weight combinations for these factors. On the other hand, in the current gas supply system risk assessment community, researchers have not made the agreement of what kind of factors or features are expected to be considered into the risk assessment model. This results in inconsistent solution directions. In this context, deep learning has been rapidly developing since the success of ImageNet [13] in the computer vision community in the past few years. As it removes the drawback of manually designed feature representation in traditional machine learning techniques, numerous industrial applications could benefit from its powerful data-processing performance, e.g., agricultural pest monitoring [14] and driver behaviour identification [15]. Thus, it is worth re-considering the gas supply system risk assessment problem from this new perspective. In this paper, we investigate the field of urban gas supply system research, then re-format and define a new task, namely Urban Natural Gas Supply System Risk Assessment (GSS-RA), to deal with the pipeline risk problem.

In general, there are three issues when employing deep-learning techniques into the urban gas supply system risk assessment system. First, it is well known that a large-scale dataset plays a key role in driving efficient models and enables powerful feature representation. When we re-define the GSS-RA task, there are few datasets targeted at solving this problem. In the field of natural gas supply systems, the major challenge lies in how to select the types of information that might be significant for risk assessment. Secondly, the various kinds of data shown in different distributions and the feature vectors or embeddings extracted from these multi-modal data might not be represented in the similar latent space. So how to design a deep-learning model that could deal with multi-modal data might be a serious issue. Finally, in the newly formatted gas supply system risk task, it is necessary to build a proper evaluation system to validate the performance of our model.

**Figure 1:**
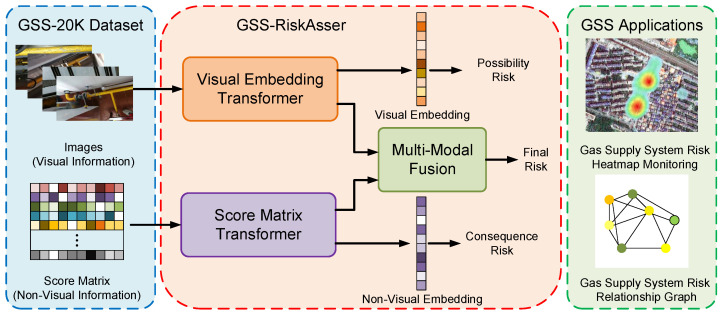
The overall pipeline of our work. To define GSS-RA task, we first build a GSS-20K dataset that contains multi-modal information, i.e., images for visual information and score matrix for non-visual information. For tackling the multi-modal problem, we design a GSS-RiskAsser that could process visual and non-visual data at the same time. Finally, our GSS-RiskAsser could be practically used in two applications: Gas Supply System Risk Heatmap Monitoring and the Gas Supply System Risk Relationship Graph.

In this paper, our main work is to solve the GSS-RA task from the above aspects. To tackle the data problem, we present a novel data collection with an analysis system. We collect information about its natural gas supply system with both visual and non-visual modals. In brief, we adopt an image–language pair as input (represented by a gas supply system image and a gas usage score matrix) that indicates the gas supply system situation for each user in our city environment. In this way, we build the first dataset for GSS-RA task, named GSS-20K. In terms of deep-learning architecture designing, we propose a novel framework GSS-RiskAsser for dealing with the multi-modal input data. In our GSS-RiskAsser, we encode the image and score matrix in two types of one-dimensional vector. Then we build a parallel transformer-like neural network to separately extract the feature embedding. To deal with the multi-modal input problem, we propose a cross-attention mechanism into these two branches that achieves multi-modal information exchange and fusion, so we can jointly obtain the final feature embedding that considers different types of information at the same time. Finally, we employ our GSS-RiskAsser into two practical applications: Gas Supply System Risk Heatmap Monitoring and User Gas Supply System Relationship Graph. We believe that our efforts could benefit future city gas supply system monitoring and warning systems. The overall pipeline of our work is illustrated in Figure 1.

The major contributions of this paper are three-fold:

- To the best of our knowledge, we are the first to consider urban gas supply system risk assessment as a deep-learning problem. We analyze and attempt to solve the challenges when employing deep-learning techniques in this field. We re-format and define a novel task Urban Natural Gas Supply System Risk Assessment (GSS-RA). To drive the model, we publish a dataset GSS-20K that uses both image and language types of data for GSS-RA task.
- Accompanying the GSS-RA task and GSS-20K dataset, we build a practical gas supply system risk assessment system, and design a parallel transformer to process image and score matrix input for each sample. To deal with the multi-modal data problem, we propose a cross-attention mechanism into these two branches that achieves multi-modal information exchange and fusion.
- To properly evaluate our work, we employ our GSS-RiskAsser into two practical applications: Gas Supply System Risk Heatmap Monitoring and User Gas Supply System Relationship Graph. We believe that our efforts provide a feasible benchmark, which serves as a strong baseline for the GSS-RA task, and in the future could facilitate research and practical applications on city gas supply system monitoring and warning. Our dataset and code will be made publicly available.

## 2. Related Work

### 2.1. Urban Natural Gas Risk Assessment

Early works for gas risk assessment consider it as a mathematical problem. On the contrary, these works try to solve this task by defining their own evaluation metrics to assess the gas risk from qualitative and quantitative aspects [16]. Jo et al. analyzes the natural gas network through the parameters of fatal length and cumulative fatal length [9]. Away from length of pipeline, Zhou et al. takes the pipeline risk with land-use planning [7]. These two works hold an obvious drawback that they provide a definitive and binary pipeline risk prediction while in practical applications we prefer the risk level or possibility rather than index. Thus, a fuzzy inference system is employed into the gas pipeline analysis system [8,17,18]. From the perspective of risk factors, Hao and You propose a bow-tie model that considers casualties, property damage, environment and society to predict the consequence [10]. Similarly, the structure and social parameters of urban blocks are also important for urban gas pipelines [19]. Moreover, Li et al. adopts a pipeline risk assessment model into a pre-warning system [11].

The methods mentioned above tend to manually build mathematical models to predict urban gas risk. With the development of artificial intelligence, more and more researchers pay their attention to constructing machine learning or neural network models. Li et al. proposes a unified framework for the new application in the vulnerability assessment of urban gas using Support Vector Machine (SVM) and Artificial Neural Network (ANN), and compares the two methods in performance [3]. In addition, Zhang and Weng exploit the Bayesian network to build a gas risk assessment model [20], in which they collect and analyze fault data as well as pipeline attributes, and establish the relationship between these variables. In terms of unsupervised methods, Wang and Li propose a method based on clustering and statistical validation by analyzing some historical fault records [21]. Although these methods could partly achieve automatic gas supply system risk assessment, they might not be considered to have enough information related to urban gas pipeline networks, e.g., visual images. In recent years, deep learning has been widely used in lots of automatic systems, for example, image processing [22], language understanding [23] or games [24]. Therefore, to the best of our knowledge, this is the first work to introduce deep-learning techniques, especially multi-modal information processing, into the urban gas supply system risk assessment field.

### 2.2. Deep Learning on Multi-Modal Representation

Multi-modal fusion is one of the key topics in multi-modal research, which aims to integrate information extracted from different modalities into a stable multi-modal representation. Currently, the total deep learning-based multi-modal representation methods could be summarized into two families. One of those is proposed to fuse information from various sensors by tensor operations, such as concatenation [25,26] or weighted sum [27]. Moreover, some works also try to employ a neural architecture search with progressive exploration [28,29] to find suitable settings for several fusion functions. Apart from tensor-based fusion operations, the other multi-modal fusion methods focus on attention-based frameworks such as Transformer [30], BERT [31] and ViT [32]. These works introduce an attention mechanism to mine the valuable information weights from a sequence query while ignoring each element’s position in input vector. Zhu et al. extend the LSTM [33] and add an image attention model based on the hidden state of the previous LSTM model for a text-processing problem [34]. Similarly, stacked attention networks (SANs) have also been proposed to use a multi-layer attention model to perform multiple queries on the image, and gradually infer the answer, simulating a multi-step reasoning process [35]. The above methods use unidirectional attention module to extract information. Anderson et al. introduces another bilateral strategy that simulates the human visual system by combining two visual attention mechanisms [36]. In recent years, benefitting from self-attention mechanism [30], researchers start to investigate Transformer or BERT-based multi-modal fusion methods, e.g., two encoder streams proposed to process visual and textual inputs separately [37] or a cross-modality encoder on higher-level proposed cross-modality features [38].

## 3. Problem Statement

As is mentioned earlier, we re-format the gas supply system risk assessment as a new GSS-RA task. In contrast to current approaches for tackling this problem, the GSS-RA problem setting advances them in these aspects: (1) we consider the gas supply system risk on a user level while most works investigate it at urban plot level. (2) we exploit both visual and non-visual information to define a multi-modal task for this problem. (3) we build a hierarchical risk evaluation system where the final gas supply system risk $Y_{r}$ is defined with possibility of gas supply system rupture $Y_{p}$ as well as predicted consequence level (severity) after gas supply system rupture $Y_{s}$.

In the GSS-RA problem, given a 2D single static RGB image *I* that describes the user’s gas supply system and a pipeline situation score matrix *M* from several experts’ judgement, a GSS-RA model will learn to predict the three gas supply system risk metrics $Y_{p}$, $Y_{s}$ and $Y_{r}$ for each user. Therefore, our GSS-RA task requires the model to deal with both visual and non-visual features and achieve multi-modal data-processing ability. Finally, to validate the practical performance of the GSS-RA task and our proposed framework, we will also employ it into two typical urban gas supply system applications.

## 4. Dataset

As few works focus on dealing with a gas supply system risk assessment with deep-learning methods and there exists no dataset to fully support our GSS-RA task, we construct the GSS-20K dataset by manual acquisition and annotation. First, we acquire gas supply system images for each user to build the visual subset (Section 4.1). Then, we collect the GSS score matrix with our proposed a new gas supply system judgement paradigm (Section 4.2). Based on both the collected visual and non-visual data, we invite experts to annotate the hierarchical risk values. Finally, due to the data imbalance problem, we propose a GSS data expansion strategy to build our large-scale GSS-20K dataset that could drive the model training (Section 4.3). We then analyze the effectiveness of our dataset (Section 4.4).

### 4.1. GSS Image Acquisition

**Figure 2:**
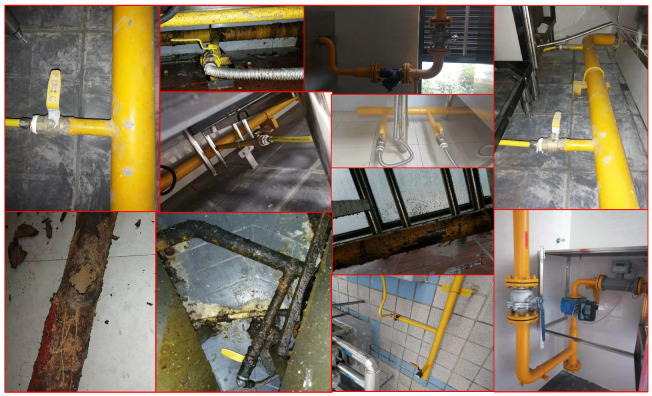
Part of image samples in GSS-20K dataset.

Current deep-learning datasets, such as MS COCO [39], usually collect images using Google or Bing. However, images from the Internet may not be suitable for building a multi-modal GSS-RA system and constructing the image–language pair in the dataset. Moreover, we may want to establish our dataset at user level. This requires us to capture RGB images at every user’s workplace or home. For this purpose, we spend nearly half a year on uniformly sampling 5000 users in our city and capture at least 10 images for each of them. We require the images to represent the visual information of the gas supply system, e.g., texture, materials, wear, and so on. Finally, during training of our network, we randomly select one of the 10 captured images at each dataset sampling. Some samples of the GSS images are shown in Figure 2.

### 4.2. GSS Score Matrix Collection

In many deep-learning tasks, such as agricultural pest detection [40], visual information could support its practical applications, so these works might only rely on image as input. But for the GSS-RA task, due to the complexity of user gas supply system environments, we may not capture structured images, which will affect the final assessment performance. Thus, we introduce a GSS score matrix as another modal datum to assist our network for precise risk assessment. Specifically, for each user, we invite at least 5 pipeline investigators and provide a score matrix considering the following two factors:

**Accident-related factors:** We first consider what factors might cause gas supply system accidents. (1) Hose defect. (2) Burning appliance defect. (3) User misconduct. (4) Pipeline and equipment defects. (4) Management. **Consequence related factors:** Secondly, we also consider the loss of life and property when an accident happens. (1) Difficulty of repair. (2) Safety interlocking device. (3) Gas environment. The details of these factors and the scoring paradigm are shown in Table 1 and Table 2.

To obtain the final score matrix, we adopt one-hot encoding function to build the score related to each factor into a 1-dimensional vector (containing 10 elements) and concatenate these vectors into our score matrix with N×10 dimensions.

### 4.3. Positive Data Generation

**Figure 3:**
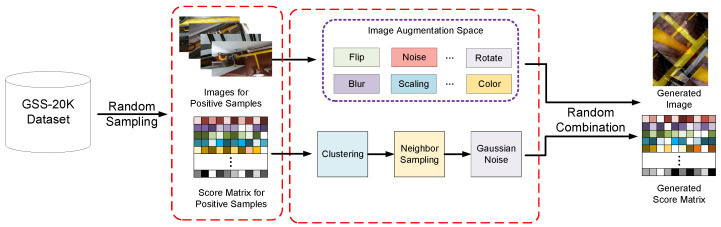
Our method for generating positive data. Due to the difficulty in collecting positive data samples, we adopt a positive data-generation strategy to expand the GSS-20K dataset and solve the data imbalance problem.

Compared with image collection for visual tasks, e.g., image classification [13], it is far more challenging to collect multi-modal data for a GSS-RA task. Moreover, due to some realistic reasons, we can only obtain a few positive samples that indicate high-risk users. Therefore, it is evident that there exists a data imbalance problem in our dataset. To deal with this, motivated by computer graphics simulation theory, we fully exploit the value of captured samples to propose a data-generation strategy for our task, named Semi-Composited Multi-Modal Generation (SCMMG). The pipeline of our SCMMG approach is shown in Figure 3.

Our SCMMG method includes the following steps. For visual data generation, we build a complex data augmentation pipeline that adopts mix-up, rotate, noise, blur and some popular strategies to generate an image that is an unseen and positive sample in the original dataset. Moreover, we also randomly sample a positive score matrix and generate a new score matrix by clustering neighbor sampling as well as Gaussian noise. Finally, we randomly combine the generated visual and non-visual information to obtain the expanded multi-modal data. In this way, we build our GSS-20K dataset with enough positive samples.

### 4.4. Comparison with Other Datasets

To further motivate the construction and usage of our dataset, we compare GSS-20K with several existing datasets from two aspects, i.e., comparison with datasets for some popular deep-learning tasks and comparison with datasets that are related to the task of gas supply system risk assessment. The comparison is illustrated in Table 3.

As can be observed, image classification and object detection are the key topics in the computer vision and deep-learning community, where many researchers participate in building large-scale datasets as their benchmarks. Thus, ImageNet [13] or MS COCO [39] hold numerous training and testing samples. As for multi-modal dataset, some works tend to fuse image and text in a dataset and provide a question-answering system [41]. In terms of employing deep learning in practical applications, IP102 [42] and Zhang’s dataset [43] supports their tasks (pest recognition and train fault detection) and prove that deep-learning models could work well when trained with enough samples. These successful cases also point out that computer vision techniques could achieve good performance in practical automatic systems. However, for the GSS-RA task that we target in this paper, only visual information seems not to be a good way. Wang et al. [21] and Li et al. [3] have tried to introduce a learning-based method to investigate risk assessment tasks, where they use a score vector or score matrix approaches rather than visual information in their works. But their data volume is too small to drive a deep-learning model. Therefore, we build the first dataset GSS-20K for our GSS-RA task.

## 5. Method

**Figure 4:**
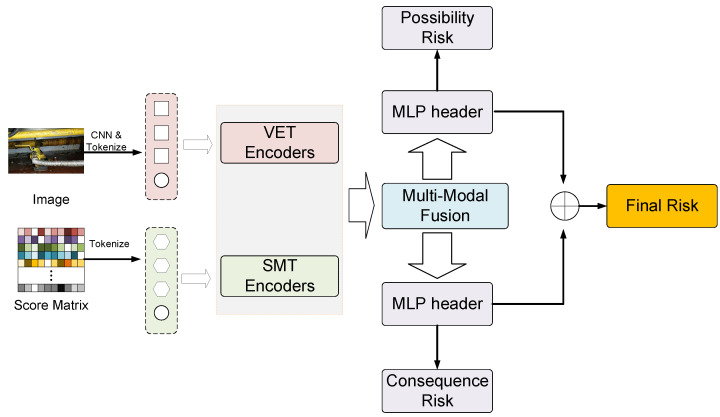
Framework Overview.

We propose a framework GSS-RiskAsser to address GSS-RA task, whose structure in Figure 4. The key challenge for our method is to handle multi-modal information processing, i.e., how to extract multi-modal features from different modal inputs separately and how to reasonably fuse them to predict final gas supply system risk. In our GSS-RiskAsser framework, the multi-modal feature extraction can be tackled by Visual Embedding Transformer (VET) in Section 5.1 and Score Matrix Transformer (SMT) in Section 5.2. And a Multi-Modal Fusion (MMF) module is also designed to combine and fuse these features for final prediction (Section 5.3).

### 5.1. Visual Embedding Transformer (VET)

The great success of transformers in NLP has sparked particular interest from the computer vision community in introducing transformer architectures into processing 2D images [30]. In our GSS-RiskAsser, we adopt a self-attention mechanism in transformers and propose a Visual Embedding Transformer (VET) to deal with our gas supply system images.

**Figure 5:**
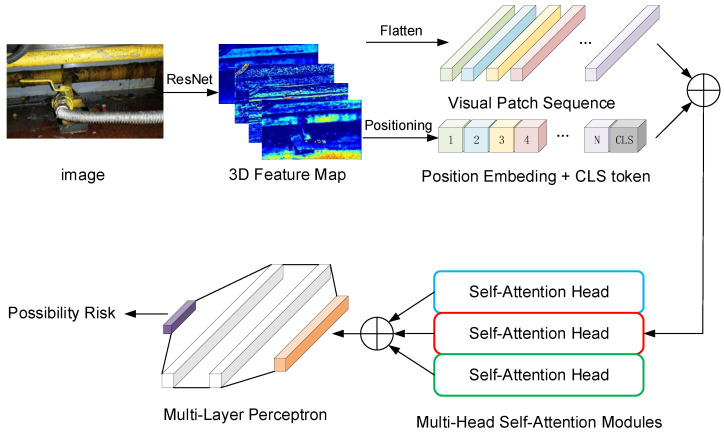
The structure of Visual Embedding Transformer.

The structure of VET is shown in Figure 5. In contrast to a standard visual transformer, we combine the transformer architecture with a Convolutional Neural Network (CNN) for pre-processing. The input image is fed into a popular ResNet [22] and output the 3D feature map with shape of W×H×C. To deal with the 3D tensor, we reshape and group it into several 2D feature map patches $\left(N\times \left(P^{2}\cdot C\right)\right)$, where $N=HW/P^{2}$ and (P,P) is the size of feature map patch. In this way, we could flatten the 3D feature map with a trainable projection and obtain a patch sequence $X^{\left(I\right)}$.

Next, given the patch sequence $X^{\left(I\right)}$, we build several transformer encoders to further refine the visual features. Specifically, we add a class token $x_{cls}$ at the beginning of sequence $X^{\left(I\right)}$ to be $\left[x_{cls};x_{I}^{1};x_{I}^{2};\ldots ;x_{I}^{N}\right]$, where $x_{I}^{k}\in R^{\left(P^{2}\cdot C\right)}$. In addition, to maintain position information of each feature patch, we encode the 1D position embedding $\left[x_{p}^{1};x_{p}^{2};\ldots ;x_{p}^{N}\right]$ and augment them into the input patch sequence so that we could obtain the input sequence into transformer encoders:

(1) $$z_{0}^{\left(I\right)}=\left[x_{cls};x_{p}^{1}x_{I}^{1};x_{p}^{2}x_{I}^{2};...;x_{p}^{N}x_{I}^{N}\right]$$

**Figure 6:**
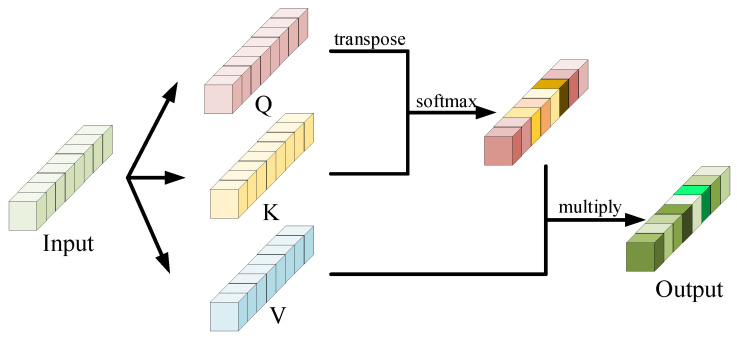
The structure of self-attention module.

Each transformer encoder is composed of a Multi-head Self-Attention (MSA) module and a Multi-Layer Perceptron (MLP). For *l*th encoder, the input sequence $z_{l-1}^{\left(I\right)}$ is first fed into a MSA module and output a processed feature sequence:

(2) $$z_{l}^{\prime (I)}=MSA\left(LN\left(z_{l-1}^{\left(I\right)}\right)\right)+z_{l-1}^{\left(I\right)}$$

where LN represents Layer Normalization operation. In detail, MSA module employs some (here we use three) parallel self-attention modules, shown in Figure 6. ’Attention’ here is to calculate the relevance for each patch pair. In each self-attention, we create three tensors $Q_{l-1}$, $K_{l-1}$ and $V_{l-1}$:

(3) $$Q_{l-1}=K_{l-1}=V_{l-1}=z_{l-1}$$

which means $Q_{l-1}$, $K_{l-1}$ and $V_{l-1}$ are copied from input $z_{l-1}$. Then, we calculate the attention vector as a weight $\alpha_{l-1}$:

(4) $$\alpha_{l-1}=\frac{e^{f_{q}\left(Q_{l-1}^{T}\right)f_{k}\left(K_{l-1}^{j}\right)}}{\sum_{j=1}^{N}e^{f_{q}\left(Q_{l-1}^{T}\right)f_{k}\left(K_{l-1}^{j}\right)}}$$

where $f_{q}\left(\cdot \right)$ and $f_{k}\left(\cdot \right)$ are two functions to pre-process $Q_{l-1}$ and $K_{l-1}$ and here we use two fully connected layers to train these two functions. Given the weight $\alpha_{l-1}$, we could obtain the self-attention output $A_{l-1}$:

(5) $$A_{l-1}=\sum_{j=1}^{N}\alpha_{l-1}^{j}V_{l-1}^{j}$$

Thus, the MSA output is the sum of these self-attention outputs. Finally, we further adopt MLP and short-cut mechanism to obtain the transformer encoder output $z_{l}$:

(6) $$z_{l}^{\left(I\right)}=MLP\left(LN\left(z_{l}^{\prime (I)}\right)\right)+z_{l}^{\prime (I)}$$

Finally, we use $z_{l}^{\left(I\right)}$ to predict the gas supply system risk $Y_{p}$ (possibility of gas supply system rupture).

### 5.2. Score Matrix Transformer (SMT)

Similar to VET, we also build a transformer-like architecture to process score matrix data, named Score Matrix Transformer (SMT). First, we flatten the score matrix into a patch sequence $X^{\left(M\right)}$. Since we collect score matrix by various gas supply system attributes, each position of patch sequence indicates each attribute. Then, we also add class token $x_{cls}$ and position embedding into it:

(7) $$z_{0}^{\left(M\right)}=\left[x_{cls};x_{p}^{1}x_{M}^{1};x_{p}^{2}x_{M}^{2};...;x_{p}^{N}x_{M}^{N}\right]$$

Next, we build several transformer encoders to process this sequence $z_{0}^{\left(M\right)}$ and compute each layer’s transformer encoder output $z_{l}^{\left(M\right)}$, which also contain MSA modules and MLPs:

(8) $$z_{l}^{\prime (M)}=MSA\left(LN\left(z_{l-1}^{\left(M\right)}\right)\right)+z_{l-1}^{\left(M\right)}$$

(9) $$z_{l}^{\left(M\right)}=MLP\left(LN\left(z_{l}^{\prime (M)}\right)\right)+z_{l}^{\prime (M)}$$

Please note that due to the fact that score matrix is not in the same distribution of images, the transformer encoders in SMT do not share any weights with those in VET. Finally, we use $z_{l}^{\left(M\right)}$ to predict the gas supply system risk $Y_{s}$ (consequence severity level after gas supply system rupture).

### 5.3. Multi-Modal Fusion (MMF)

**Figure 7:**
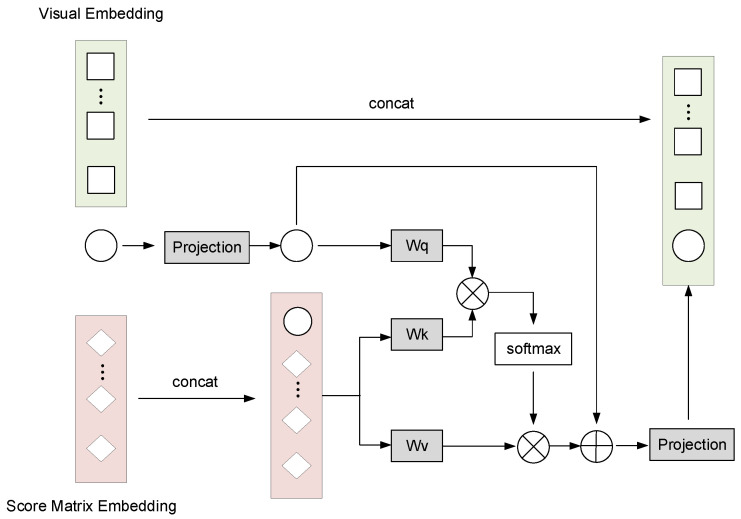
The structure of Multi-Modal Fusion.

In GSS-RiskAsser, we aim to tackle the multi-modal information problem. Therefore, we design a Multi-Modal Fusion (MMF) module to achieve reasonable and effective feature fusion from VET and SMT. The architecture of MMF is shown in Figure 7. As can be observed, we employ cross-attention mechanism for this purpose. Specifically, we first use the CLS token $x_{cls}$ at VET or SMT branch as an agent to exchange information among the patch sequence from the other branch and then back-project it to its branch. During the information exchanging, the patch sequence could be refined by $x_{cls}$. On the other hand, after the fusion with the patch sequence, the $x_{cls}$ could interact with its own patch sequence at its transformer encoder, so that the information from other modalities would enrich the features. Therefore, the features from VET and SMT could be well fused.

Taking the CLS token $x_{cls}^{\left(I\right)}$ in VET branch and patch sequence $z_{l}^{\left(M\right)}$ in SMT branch as example, our MMF module will first concatenate them:

(10) $$z_{0}^{\left(VET\right)}=[f^{\left(I\right)}\left(x_{cls}^{\left(I\right)}\right)\left|\right|z_{l}^{\left(M\right)}]$$

where $f^{\left(I\right)}\left(\cdot \right)$ is the projection function for dimensions alignment. Then we perform cross-attention mechanism between $x_{cls}^{\left(I\right)}$ and $z_{0}^{\left(VET\right)}$. Mathematically, for *l*th layer, the Query $Q_{l-1}$, Key $K_{l-1}$ and Value $V_{l-1}$ could be computed by:

(11) $$\begin{array}{c} Q_{l-1}=x_{cls}^{\left(I\right)}W_{l-1}^{q}\\ K_{l-1}=z_{0}^{\left(VET\right)}W_{l-1}^{k}\\ V_{l-1}=z_{0}^{\left(VET\right)}W_{l-1}^{v} \end{array}$$

where $W_{l-1}^{q}$, $W_{l-1}^{k}$ and $W_{l-1}^{v}$ are trainable parameters. Then we apply self-attention to obtain the weight vector $\alpha_{l-1}^{j(F)}$ by:

(12) $$\alpha_{l-1}^{j(VET)}=\frac{e^{Q_{l-1}^{T}K_{l-1}^{j}}}{\sum_{j=1}^{N}e^{Q_{l-1}^{T}K_{l-1}^{j}}}$$

(13) $$A_{l-1}^{\left(VET\right)}=\sum_{j=1}^{N}\alpha_{l-1}^{j(VET)}V_{l-1}^{j}$$

In this way, by combining several cross-attention modules, we could build a Multi-head Cross-Attention (MCA) module to compute the final fusion output at *l*th layer on VET branch:

(14) $$z_{l}^{\left(VET\right)}=[g^{\left(I\right)}\left(y_{cls}^{\left(I\right)}\right)\left|\right|z_{l}^{\left(I\right)}]$$

where $g^{I}\left(\cdot \right)$ is the projection and back-projection function for dimension alignment. $y_{cls}^{\left(I\right)}$ could be computed by:

(15) $$y_{cls}^{\left(I\right)}=MCA(LN([x_{cls}^{\left(I\right)}\left|\right|z_{l}^{\left(M\right)}]))+x_{cls}^{\left(I\right)}$$

In the same way, we could obtain the output for SMT $z_{l}^{\left(SMT\right)}$. Finally, we use $z_{l}^{\left(F\right)}=[z_{l}^{\left(VET\right)}\left|\right|z_{l}^{\left(SMT\right)}]$ to predict the final gas supply system risk $Y_{r}$.

## 6. Experiments

### 6.1. Experiment Settings

**Implementation Details** We train our model for 300 epochs on 8 Titan X GPUs with a batch size of 1024. In terms of optimizer, we use cosine linear-rate scheduler with the initial learning rate of 0.001 and a weight decay of 0.005. During training and inference, we resize all the images into 224×224 resolution as the input. Our code is based on Python and PyTorch deep-learning framework.

**Baselines** Since GSS-RA is a novel task and no existing baseline methods could work in this task. We compare our GSS-RiskAsser with the following popular deep-learning models: (1) CNN-based methods. We only apply CNN architecture to process visual information and predict gas supply system risk. The models we compare are ResNet50 and ResNet101. (2) RNN-based methods. In these baselines, we only exploit score matrix by DNN, GRU or LSTM to predict risk value. (3) CNN-RNN fusion methods. We extract multi-modal features by CNN and RNN separately and concatenate them to predict risk. Apart from these methods, we also compare our GSS-RiskAsser with its ablated versions such as VET only and SMT only. Moreover, we report ablation studies on various GSS-RiskAsser versions, e.g., single-head self-attention, single attention for multi-modal fusion and different image/patch sizes.

**Evaluation Metrics** For three different types of risks, we use Mean Absolute Error (MAE) and Mean Square Error (MSE) as evaluation metrics.

(16) $$\begin{array}{c} MAE\left(Y,\hat{Y}\right)=\frac{1}{N}\sum_{i=1}^{N}\left|y_{i}-{\hat{y}}_{i}\right|\\ MSE\left(Y,\hat{Y}\right)=\frac{1}{N}\sum_{i=1}^{N}{\left(y_{i}-{\hat{y}}_{i}\right)}^{2} \end{array}$$

where *N* is the number of testing samples. $y_{i}$ and ${\hat{y}}_{i}$ indicate the predicted risk value and ground truth risk, respectively. Generally, MAE measures the risk assessment accuracy while MSE measures the robustness of the estimates. Moreover, we also adopt accuracy at various risk error threshold as the metric.

### 6.2. GSS-RiskAsser Performance

Table 4 shows the urban gas supply system risk assessment results under different methods. Generally, we can see that multi-modal methods that adopt both visual and non-visual information significantly outperform single-modal methods on three types of risk metrics. In detail, DNN is not a good baseline in our dataset, which only builds several MLPs to predict risks. Moreover, Vision Transformer [32] might achieve only a slight improvement compared to ResNet50 and ResNet101, which indicates that visual information is not enough in our GSS-RA task. On the other hand, when obtaining multi-modal inputs, the methods could obtain a large margin on possibility risk assessment. This could be explained by score matrix, where multi-modal methods could more efficiently extract visual features for risk assessment. This phenomenon seems to be much more pronounced on our GSS-RiskAsser, which perform **0.054**, **0.040** and **0.036** MAE on the possibility risk, consequence risk and final risk assessment. Therefore, we can conclude that multi-modal features could play a complementary role in our task.

**Figure 8:**
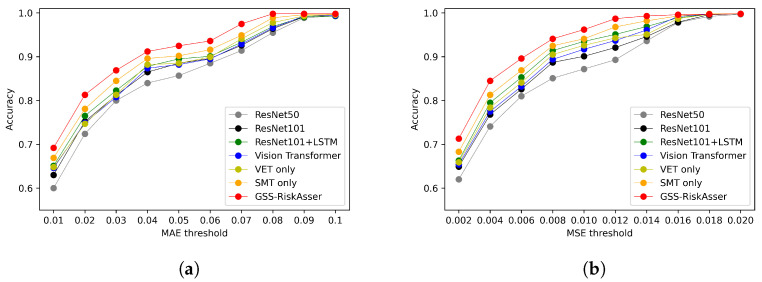
Accuracy under various MAE and MSE threshold for possibility risk assessment comparison on GSS-20K dataset. (**a**) Accuracy under MAE threshold. (**b**) Accuracy under MSE threshold.

**Figure 9:**
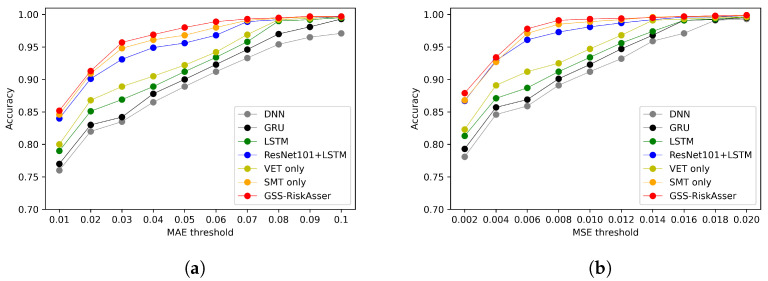
Accuracy under various MAE and MSE threshold for consequence risk assessment comparison on GSS-20K dataset. (**a**) Accuracy under MAE threshold. (**b**) Accuracy under MSE threshold.

**Figure 10:**
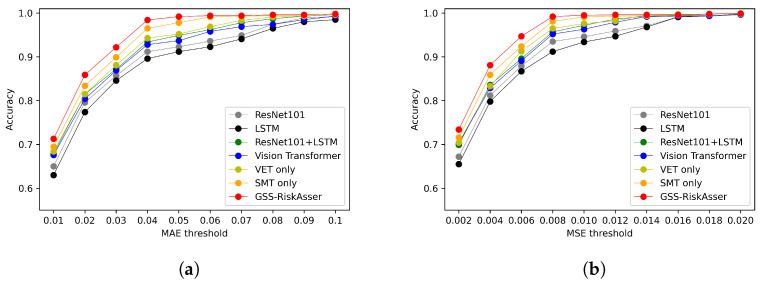
Accuracy under various MAE and MSE threshold for final risk assessment comparison on GSS-20K dataset. (**a**) Accuracy under MAE threshold. (**b**) Accuracy under MSE threshold.

We also report accuracy on various MAE and MSE thresholds. Figure 8, Figure 9 and Figure 10 illustrate the accuracy curves on possibility risk, consequence risk and final risk. As can be observed, most methods could obtain satisfied performance on around MAE 0.2 and MSE 0.03. However, under more stringent thresholds, the accuracy might achieve poorer results that are around **70%**, **80%** and **70%** on three types of risks in MAE. Among these accuracy curves, our GSS-RiskAsser is the best method and obtain a larger improvement compared to others.

### 6.3. Ablation Studies

We built some ablation experiments on investigating the effects of architecture parameters, fusion strategy and image/patch sizes. In these experiments, we use MAE as metrics and set several different parameter combinations.

**Effect of architecture parameters.**Table 5 illustrates the different parameters of our GSS-RiskAsser on GSS-20K dataset. In GSS-RiskAsser, there are three major modules of VET, SMT and MMF, which contains different number of encoders, respectively. In Table 5, we use M, K, and L to indicate the three encoders. As it can be seen, number of VET encoders is a key parameter that largely influence the performance of GSS-RiskAsser. From 2 to 4, the MAE drops from **0.065** to **0.055** on MAE of possibility risk. On the contrary, our method is more robust to the number of SMT encoders, in which there is only around **0.001** decline. This could be attributed to the reason that score matrix input is already in a high semantic space so SMT could easily learn the high-quality features from it.

**Effect of fusion strategy.** As discussed earlier, multi-modal information fusion is one of the major challenges in processing different types of features. In Table 6, we investigate the effect of different fusion strategy, where we experiment with some common fusion methods. We can see that our cross-attention fusion is the best fusion strategy among these methods, which obtains **0.007** and **0.006** MAE improvements compared to concatenation fusion and sum fusion. In addition, under the multi-level cross-attention strategy, our GSS-RiskAsser could achieve higher performance on our task.

**Effect of image/patch sizes.** In GSS-RiskAsser, we use image as visual information input, so here we discuss how to resize the image shape and how to tokenize the image into patch sequence. In Table 7, we report the results on various image sizes and patch sizes. It is obvious that higher image resolution leads to higher risk assessment performance, where 480×480 is the best image input resolution. In terms of patch size, there is not a very apparent difference when using 12 patches and 16 patches. We can explain this phenomenon by the fact that VET encoders could process image patch sequences well and do not require a long-length sequence.

### 6.4. Practical Applications Using GSS-RiskAsser

**Figure 11:**
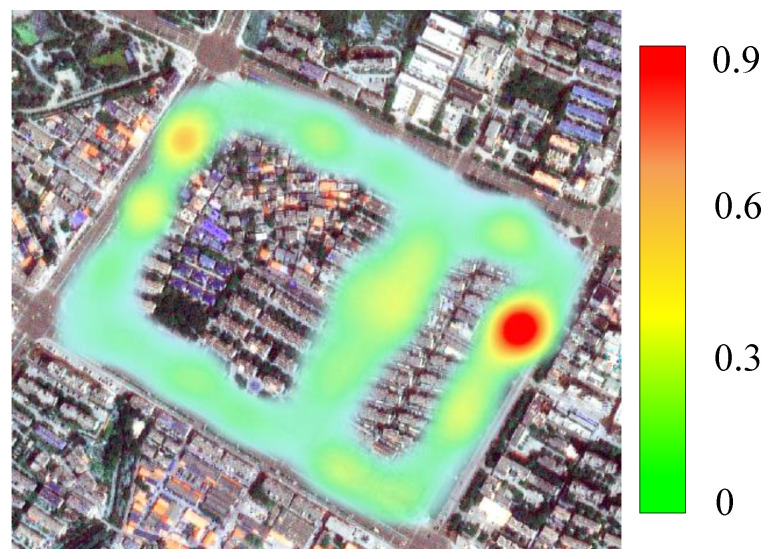
Gas Supply System Risk Heatmap Monitoring. Please note that the business users are usually distributed along the street and here we only draw the gas supply system risk for every business user.

**Gas Supply System Risk Heatmap Monitoring.** We deploy our GSS-RiskAsser into practical urban natural gas pipeline applications. The first one is gas supply system risk heatmap monitoring, as shwon in Figure 11. In this application, we survey and collect gas supply system images as well as score matrix as described in Section 4.4, and then drive our GSS-RiskAsser to predict each user’s risk value. In this way, we can monitor the users gas supply system risk distribution of the whole city. We believe this application could help to provide scientific guidance for management decision-making and reduce the possibility of pipeline rupture accidents. We can see the distribution of business users’ risks in Figure 11. It proves that with the help of our GSS-RiskAsser system, we can monitor every urban block’s gas supply system risk and could guide the repairing and warning solution.

**Figure 12:**
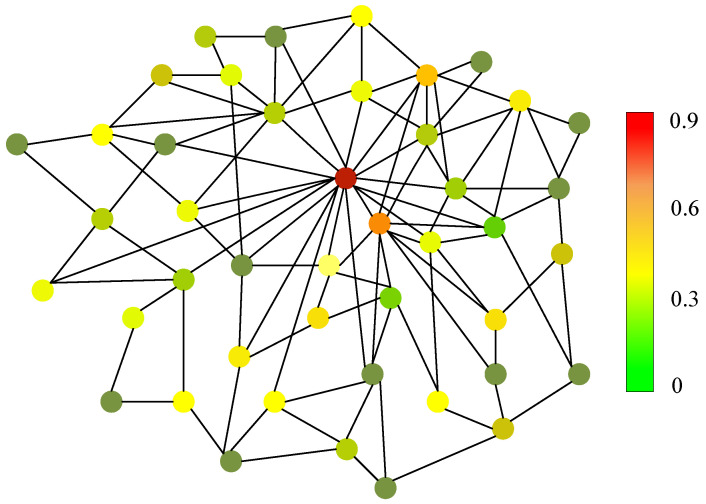
Gas Supply System Risk Relationship Graph. Please note that we draw the graph for best view so that the geometric positions in this figure are not corresponding to their actual positions in urban blocks.

**Gas Supply System Risk Relationship Graph.** To further investigate the gas supply system risk distribution as well as the potential accident reasons. We deploy our GSS-RiskAsser and draw the gas supply system risk relationship graph in Figure 12. In this application, we illustrate each user’s risk and their relationship. The links in this graph represent geometric distance of these users. As it can be seen, every user’s gas supply system is partly influenced by its neighbors in this figure. In this way, we could further mine the relationship between the gas supply system risk and the corresponding users’ locations. Therefore, we can infer when one user might meet high risk, its neighbor might also be influenced. In addition, from this figure, we can be also guided to correctly inference the interrelation of gas supply system risks among the business users.

## 7. Conclusions

In this work, we study the urban gas supply system risk assessment problem from a deep-learning perspective, and define a novel task GSS-RA. To further investigate the GSS-RA task and provide a proper method as baseline, we collect and build a domain-specific dataset GSS-20K that contains multi-modal data. To the best of our knowledge, we are the first to adopt a deep-learning approach to this problem and publish the corresponding dataset. In addition, we propose a new gas supply system risk assessment framework, and design parallel transformers to process multi-modal information at the same time. To properly fuse them, we propose a cross-attention mechanism to achieve multi-modal information exchange and fusion. Experiments show that our method could work well on the GSS-RA task and in the future could promote practical applications in the real world.

## Figures and Tables

**Table 1:** Details of accident-related gas supply system factors considered in our GSS score matrix.

Factor Type	Value Intervals				Description
	I	II	III	IV	
Indoor gas supply system	[0, 0.4)	[0.4, 0.6)	[0.6, 0.8)	[0.8, 1)	The integrity and corrosion of the indoor gas equipment
Gas equipment connection pipe	[0, 0.3)	[0.3, 0.5)	[0.5, 0.7)	[0.7, 0.9)	Material, length and other status of the gas equipment
Habits and skills of using gas	[0, 0.4)	[0.4, 0.6)	[0.6, 0.8)	[0.8, 1)	Whether gas users have been trained and standardized
Number of gas equipment	[0, 0.3)	[0.3, 0.5)	[0.5, 0.7)	[0.7, 0.9)	The number of gas equipment of the user
Gas equipment quality	[0, 0.3)	[0.3, 0.5)	[0.5, 0.7)	[0.7, 0.9)	Years of gas equipment, is there a flameout protection device, etc
Environment of the gas equipment	[0, 0.2)	[0.2, 0.4)	[0.4, 0.6)	[0.6, 1)	Sanitation, ventilation and other conditions of the gas equipment
Gas use-side management	[0, 0.3)	[0.3, 0.5)	[0.5, 0.7)	[0.7, 0.9)	formulated management rules and regular inspection
Gas supply-side management	[0, 0.2)	[0.2, 0.4)	[0.4, 0.6)	[0.6, 1)	Frequency of gas company safety inspections

**Table 2:** Details of consequence related gas supply system factors considered in our GSS score matrix.

Factor Type	Value Intervals				Description
	I	II	III	IV	
Distance from the accident point	[0, 0.4)	[0.4, 0.6)	[0.6, 0.8)	[0.8, 1)	Distance of the rescue point from the accident occurrence point
Gas safety facilities	[0, 0.4)	[0.4, 0.6)	[0.6, 0.8)	[0.8, 1)	Setting of the gas leakage alarm and the emergency cut-off valve
Gas-use environment	[0, 0.6)	[0.6, 0.7)	[0.7, 0.8)	[0.8, 0.9)	Indoor ventilation conditions, the opening of doors and windows
Gas user type	[0, 0.4)	[0.4, 0.6)	[0.6, 0.8)	[0.8, 1)	kind of users, hotels, schools, hospitals and so on

**Table 3:** Comparison with Other datasets. We compare our GSS-20K dataset with the datasets for computer vision tasks, as well as those for risk assessment task.

Dataset	Task	#Samples	Multi-Modal Information	Drive for Deep Learning	Hierarchical Validation
ImageNet [13]	Image Classification	>10,000 K	-	✓	-
MS COCO [39]	Object Detection	123 K	-	✓	
VQAv2 [41]	Visual Question Answering	400 K	✓	✓	-
IP102 [42]	Pest Recognition	75 K	-	✓	✓
Zhang et al. [43]	Train Fault Detection	3.8 K	-	✓	-
Wang and Li [21]	Risk Assessment	0.7 K	-	-	-
Li et al. [3]	Risk Assessment	2 K	-	-	✓
GSS-20K (Ours)	GSS-RA	20K	✓	✓	✓

**Table 4:** Gas supply system risk assessment results on GSS-20K dataset for different methods. We compare our GSS-RiskAsser with two risk assessment families: single-modal methods that use only image or score matrix as input and multi-modal methods that use both image and score matrix for prediction. ’mean’ and ’std’ represent average and standard deviation, respectively. Please note that some single-modal methods could predict possibility risk or consequence risk due to they can only process images or score matrix as input in their methods.

Method	Possibility Risk		Consequence Risk		Final Risk	
	MAE	MSE	MAE	MSE	MAE	MSE
*Single-Modal Methods*						
DNN	-	-	0.067	0.013	0.081	0.026
ResNet50 [22]	0.086	0.019	-	-	0.069	0.012
ResNet101 [22]	0.085	0.017	-	-	0.067	0.013
GRU [44]	-	-	0.065	0.012	0.078	0.020
LSTM [33]	-	-	0.066	0.012	0.076	0.019
Vision Transformer [32]	0.085	0.016	-	-	0.066	0.012
*Multi-Modal Methods*						
ResNet50 [22] + GRU [44]	0.078	0.017	0.060	0.010	0.062	0.011
ResNet50 [22] + LSTM [33]	0.078	0.017	0.062	0.011	0.063	0.011
ResNet101 [22] + DNN	0.076	0.016	0.063	0.011	0.072	0.012
ResNet101 [22] + GRU [44]	0.074	0.015	0.058	0.009	0.054	0.008
ResNet101 [22] + LSTM [33]	0.069	0.013	0.052	0.008	0.048	0.006
*Our Methods*						
VET only	0.063	0.011	0.061	0.010	0.047	0.006
SMT only	0.072	0.015	0.045	0.006	0.042	0.004
GSS-RiskAsser	**0.054**	**0.009**	**0.040**	**0.003**	**0.036**	**0.003**

**Table 5:** Ablation Study with different architecture parameters on GSS-20K. We investigate the effects of: number of VET encoders represented by ’M’, number of SMT encoders represented by ’K’ and number of MMF encoders represented by ’L’

Method	M	K	L	MAE		
				Possibility Risk	Consequence Risk	Final Risk
GSS-RiskAsser	2	1	1	0.065	0.051	0.048
	3	1	1	0.061	0.047	0.043
	4	1	1	0.058	0.043	0.040
	4	2	1	0.055	0.042	0.038
	4	1	2	**0.054**	**0.040**	**0.036**
	4	2	2	0.056	0.041	0.038

**Table 6:** Ablation Study with different multi-modal fusion strategy on GSS-20K. We investigate the effects of: using concatenation fusion, sum fusion single-level cross-attention and our multi-level cross-attention fusion in MMF.

Method	Fusion	MAE		
		Possibility Risk	Consequence Risk	Final Risk
GSS-RiskAsser	ConcatFusion	0.065	0.051	0.047
	SumFusion	0.063	0.049	0.046
	Single-level Cross-Attention	0.057	0.043	0.040
	Mingle-level Cross-Attention	**0.054**	**0.040**	**0.036**

**Table 7:** Ablation Study with different image input size and patch size.

Method	Image Size	Patch Size	MAE		
			Possibility Risk	Consequence Risk	Final Risk
GSS-RiskAsser	224×224	8	0.067	0.049	0.046
	224×224	12	0.064	0.048	0.044
	384×384	12	0.059	0.044	0.038
	384×384	16	0.058	0.043	0.039
	480×480	12	0.055	**0.039**	0.038
	480×480	16	**0.054**	0.040	**0.036**
